# High Serum Elafin Prediction of Poor Prognosis of Locoregional Esophageal Squamous Cell Carcinoma

**DOI:** 10.3390/cancers13123082

**Published:** 2021-06-21

**Authors:** I-Chen Wu, Yao-Kuang Wang, Yi-Hsun Chen, Chun-Chieh Wu, Meng-Chieh Wu, Wei-Chung Chen, Wen-Lun Wang, Hung-Shun Lin, Chou-Cheng Chen, Shah-Hwa Chou, Yu-Peng Liu, Ming-Tsang Wu

**Affiliations:** 1 Division of Gastroenterology, Department of Internal Medicine, Kaohsiung Medical University Hospital, Kaohsiung 807, Taiwan; minica@kmu.edu.tw (I.-C.W.); 970395@kmuh.org.tw (Y.-K.W.); 1020420@kmuh.org.tw (Y.-H.C.); 2 Department of Medicine, Faculty of Medicine, College of Medicine, Kaohsiung Medical University, Kaohsiung 807, Taiwan; wucchieh@kmu.edu.tw (C.-C.W.); shhwch@kmu.edu.tw (S.-H.C.); 3 Department of Pathology, Kaohsiung Medical University Hospital, Kaohsiung Medical University, Kaohsiung 807, Taiwan; 4 Department of Internal Medicine, Kaohsiung Municipal Ta-Tung Hospital, Kaohsiung 807, Taiwan; 930293@mail.kmuh.org.tw; 5 Ph.D. Program in Environmental and Occupational Medicine, Kaohsiung Medical University, Kaohsiung 807, Taiwan; u103803001@kmu.edu.tw (W.-C.C.); new.purple@ctbc.edu.tw (C.-C.C.); 6 Research Center for Environmental Medicine, Kaohsiung Medical University, Kaohsiung 807, Taiwan; 7 Division of Gastroenterology and Hepatology, Department of Internal Medicine, E-DA Hospital/I-Shou University, Kaohsiung 824, Taiwan; ed102807@edah.org.tw; 8 Department of Laboratory Medicine & Department of Research, Education & Training, Kaohsiung Municipal Siaogang Hospital, Kaohsiung Medical University, Kaohsiung 807, Taiwan; 930570@kmhk.org.tw; 9 Department of Public Health, Kaohsiung Medical University, Kaohsiung 807, Taiwan; 10 Division of Chest Surgery, Department of Surgery, Kaohsiung Medical University Hospital, Kaohsiung Medical University, Kaohsiung 807, Taiwan; 11 Graduate Institute of Clinical Medicine, Kaohsiung Medical University, Kaohsiung 807, Taiwan; 12 Department of Family Medicine, Kaohsiung Medical University Hospital, Kaohsiung Medical University, Kaohsiung 807, Taiwan; 13 Rapid Screening Research Center for Toxicology and Biomedicine, National Sun Yat-Sen University, Kaohsiung 807, Taiwan

**Keywords:** esophageal squamous cell carcinoma, elafin, PI3, circulating markers

## Abstract

**Simple Summary:**

Conventional serum markers such as carcinoembryonic antigen (CEA), squamous cell carcinoma antigen (SCC), and tissue polypeptide antigen (TPA) have a low sensitivity in predicting the prognosis of locoregional esophageal squamous cell carcinoma cell (ESCC). In our clinical study, we found high serum elafin to be an independent outcome predictor for stage I-IIIA ESCC, considering T, N, overall stage, and treatment. In vitro experiments showed that adding recombinant elafin drove ESCC cell proliferation, migration and invasion, while shRNA attenuated elafin levels, abrogating those effects. Our results suggested serum elafin might be a noninvasive biomarker to predict the outcome of locoregional ESCC and could potentially be used as a therapeutic target.

**Abstract:**

Esophageal squamous cell carcinoma (ESCC) is a highly aggressive tumor known to have locally advanced and metastatic features which cause a dismal prognosis. We sought to determine whether elafin, a non-invasive and secretory small-molecule marker, could be used to predict prognosis in locoregional ESCC patients in human and in vitro studies. In our human study, 119 subjects were identified as having incident and pathologically-proved ESCC with stage I-IIIA tumors from southern Taiwan between 2000 and 2016. We measured their serum elafin levels at baseline and followed them until the date of cancer death or until January 2020, the end of this study. Those with high serum elafin levels were found to have a 1.99-fold risk (95% confidence interval: 1.17–3.38) shorter survival than those who did not. In our in vitro experiments, elevated elafin levels were found to drive ESCC cell proliferation, migration and invasion, while attenuation of elafin level by shRNA abrogated those effects. We concluded that elafin promotes ESCC motility and invasion and leads to a worse clinical prognosis in ESCC patients without distant metastasis.

## 1. Introduction

Esophageal cancer was the seventh most prevalent cancer and the sixth most important cause of cancer mortality worldwide in 2018 [1]. This highly aggressive tumor is known to usually have a dismal prognosis [2,3]. In Asian countries, including Taiwan, its predominant histological subtype is esophageal squamous cell carcinoma (ESCC), accounting for more than 90% of all esophageal cancer cases there. Despite recent advances in diagnosis and treatment, its prognosis remains poor, with a 5-year survival rate ranging from 15%–25%, in Taiwan 16.5% [2,4]. Thus, it would be very beneficial clinically to find a potential noninvasive clinical biomarker that could be used to predict the survival of ESCC, especially in its early stage, locoregional ESCC.

Elafin, also known as peptidase inhibitor 3 (PI3) or skin-derived anti-leukoproteinase (SKALP), is a secretory small molecule [5,6]. It is produced by epithelial cells in response to macrophage infiltration, the release of proteolytic enzymes, and disruption of epithelial integrity [7,8,9]. Although elafin mRNA or protein is known to be overexpressed in cancer tissues arising from normal squamous epithelium of head and neck, esophagus, and bronchi, when compared to adjacent normal tissues [5,6,10], there have been no studies, to our best knowledge, to examine the possible predictive or prognostic ability of its expression in locoregional ESCC patients (stage I-IIIA). To find out, we identified patients found to have locoregional ESCC at two medical centers, measured their baseline serum elafin, and followed them until cancer death or the end of the study. We also performed an in vitro study to measure the effect of elafin expression on cell motility and invasion.

## 2. Materials and Methods

### 2.1. Human Study

#### 2.1.1. Study Subjects

Study subjects were incident and pathologically-proved ESCC patients recruited from Kaohsiung Medical University Hospital (KMUH) and Kaohsiung Veterans General Hospital (KVGH), two medical centers located in southern Taiwan between 2000 and 2016. Based on criteria established by the American Joint Committee on Cancer (AJCC) 7th edition of the tumor-node-metastasis (TNM) cancer staging system, we only included patients with stage I-IIIA (T1b-4aN-/+M0) diseases for this study. All of the eligible study patients were interviewed to collect information on demographic characteristics using a standard questionnaire. Clinical information, such as cancer stage and treatment modalities, was obtained by chart review. Blood samples were obtained before any cancer treatment. The serum specimens were stored in a −80 °C freezer until analyses. This study was approved by the Institutional Review Board (IRB) of KMUH (KMUH-IRB-990354) on 14 July 2011. All participants signed the informed consent forms.

#### 2.1.2. Serum Elafin Levels by Enzyme-Linked Immunosorbent Assay (ELISA)

Serum elafin protein levels were measured by the ELISA according to the manufacturer’s instructions (Cat. ARG81504; RayBiotech, Inc., Peachtree Corners, GA, USA). A monoclonal antibody specific for elafin was pre-coated onto a microplate (Cat. P19957; RayBiotech, Inc., USA). Standards and samples were pipetted into the wells, and elafin was bound by the immobilized antibody. After washing away unbound substances, we added an enzyme-linked monoclonal antibody specific for elafin to the wells. Following a wash to remove the unbound antibody-enzyme reagent, a substrate solution was added to the wells. The color developed in proportion to the amount of elafin bound in the initial step. The intensity of the color development was measured using a microplate reader at 450 nm with a wavelength correction at 540 nm. Each assay was repeated twice, and all of the repeated results were within 10%. We used the mean of the repeated data in our final analysis. One technician (HS Lin), blinded to the clinical staging of ESCC patients, performed the measurements.

### 2.2. In Vitro Study

#### 2.2.1. Cell Lines and Cell Culture

CE48T, CE146T, CE81T2, KYSE270, and OE21 cell lines were obtained from the Food Industry Research and Development Institute (Hsinchu, Taiwan). CE48T, CE146T, and CE81T2 and its more aggressive subline (CE81T2-4), established in our previous study [11], were maintained in Dulbecco’s Modified Eagle Medium (DMEM) supplemented with 10% fetal bovine serum (FBS), 1% non-essential amino acids (NEAA), 100 U/mL penicillin and 100 μg/mL streptomycin. KYSE270 cells were cultured in DMEM/F-12 medium supplemented with 2% FBS, 100 U/mL penicillin and 100 μg/mL streptomycin, whereas OE21 cells were cultured in RPMI-1640 medium supplemented with 10% FBS, 100 U/mL penicillin and 100 μg/mL streptomycin. All the cultured products were purchased from Thermo Fisher Scientific, Inc. (Waltham, MA, USA). The cell lines were kept in a humidified incubator under 5% CO2 at 37 °C.

To increase the elafin level in culture media, full-length recombinant human elafin (rElafin) was used at a concentration of 1 ng/mL. rElafin was purchased from OriGene Technologies, Inc. (Cat. TP303136, Rockville, MD, USA).

#### 2.2.2. Elafin Knockdown by shRNA

To neutralize elafin expression, eight short hairpin RNAs, including TRCN0000073663, TRCN0000073664, TRCN0000073665, TRCN0000073666, TRCN0000073667, TRCN0000373198, TRCN0000373199, and TRCN0000373200, were purchased and transformed into CE81T2-4 (National RNAi Core Facility at Academia Sinica, Taipei, Taiwan). The lentivirus particles were prepared by co-transfection of the gene-expressing or shRNA lentiviral plasmids with spAX2 and pMD2G plasmids into HEK293T cells. Infection of cells was carried out in the presence of 10 μg/mL polybrene. After 48 h infection, the stable clones were selected with puromycin for another 48 h. The TRCN0000073663 and TRCN0000373198 strains, named Elafin shRNA#1 and Elafin shRNA#2, were found to have the highest RNA and protein suppression efficiency and were thus used in the following experiments.

#### 2.2.3. Cell Motility and Invasion Assays

Migration and invasion assays were conducted in 24-well Corning hanging inserts (Cat. 3422, Corning Incorporated, Corning, NY, USA) and 24-well BioCoat Matrigel Invasion Chambers (Cat. 354480; Corning Incorporated, Corning, NY, USA), respectively. Cells resuspended in 300 μL serum-free medium were added to the top chamber (1 × 10^5^ cells/well), and medium supplemented with DMEM/10% FBS was added to the bottom chamber as a chemoattractant. After 16–18 h of incubation at 37 °C, cells that had migrated or invaded through the membrane (migration) or Matrigel (invasion) were fixed and stained with 0.1% Crystal violet (Cat. C0775; Sigma-Aldrich, St. Louis, MO, USA). The number of cells was counted in three random fields under the 100× objective lens.

Cell migration was determined by wound healing analyzed using IBIDI Culture-Inserts. Cells (3 × 10^5^ cells/70 μL/well) were seeded into each well of a 24-well tray containing culture inserts (Cat. 80241; Ibidi GmbH, Gräfelfing, Germany). The culture inserts were removed. Cell debris was removed by washing with PBS, and then the cells were re-cultured. Images were captured and measured after wounding, calculating the distance migrated by the cell monolayer to close the wounded area at 0-, 16-, and 48-h time points. The wound-closure rate was defined as recovered distance divided by the original width of the scratch.

#### 2.2.4. Immunoblot Analysis

All cell lysates were quantified and resolved on a sodium dodecyl sulfate–polyacrylamide gel electrophoresis (SDS–PAGE) gel, transferred onto a polyvinylidene fluoride membrane (Cat. 88518; Millipore, Burlington, MA, USA). The protein markers were PageRuler™ Prestained Protein Ladder (Cat. 26616; Thermo Fisher Scientific, Waltham, MA, USA) and Novex Sharp Pre-Stained Protein Standard (Cat. LC5800; Thermo Fisher Scientific, Waltham, MA, USA). The membranes were incubated with the indicated primary antibodies, followed by horseradish peroxidase-conjugated secondary antibodies (1:20,000; anti-mouse: Cat. 115-035-003; anti-rabbit: Cat. 111-035-003; Jackson ImmunoResearch, West Grove, PA, USA) and an enhanced chemiluminescence solution (NEN Life Science, Boston, MA, USA). The following antibodies were used: Elafin (1:500; Cat. HM2063; Hycult Biotech, Wayne, PA, USA) and β-actin (1:2000; Cat. 4970; Cell signaling Technology, Danvers, MA, USA). The signal of indicated proteins was developed on RX-N X-ray film (FUJIFILM Corporation, Tokyo, Japan)

### 2.3. Statistical Analysis

Human and in vitro studies were analyzed using analysis of variance (ANOVA) or Student *t*-test for continuous variables or by the χ^2^ test for categorical variables, where appropriate. For the human study, Kaplan–Meier analysis with log-rank testing was used to examine whether serum elafin levels predicted survival. Each participant accumulated person-time beginning from the date of ESCC diagnosis and ending on the date of cancer death or the end of this study in January 2018. Serum elafin levels were divided into tertiles from 1st (lowest) to 2nd and 3rd (highest) and also dichotomized by the combination of 2nd and 3rd tertiles compared to the 1st one. Cox proportional hazards models were used to compute hazard ratio (HR) and 95% confidence interval (CI) and control for other covariates. The covariates in Cox regression included age, sex, stage, and treatment. All statistical operations were performed using the SAS Version 9.3 (Cary, NC, USA) statistical software package; a *p*-value of less than 0.05 was considered significant.

## 3. Results

### 3.1. Human Study

The 119 patients with stage I-IIIA ESCC had an average age of 57.83 years old (range: 38–82) and were predominantly male (95.3%) (Table 1). The mean serum elafin levels were 80.34 ± 88.06 ng/mL (range 2.70–502.83). The only significant differences among the serum tertile groups were age, stage, and treatment (Table 2).

**Figure 1:**
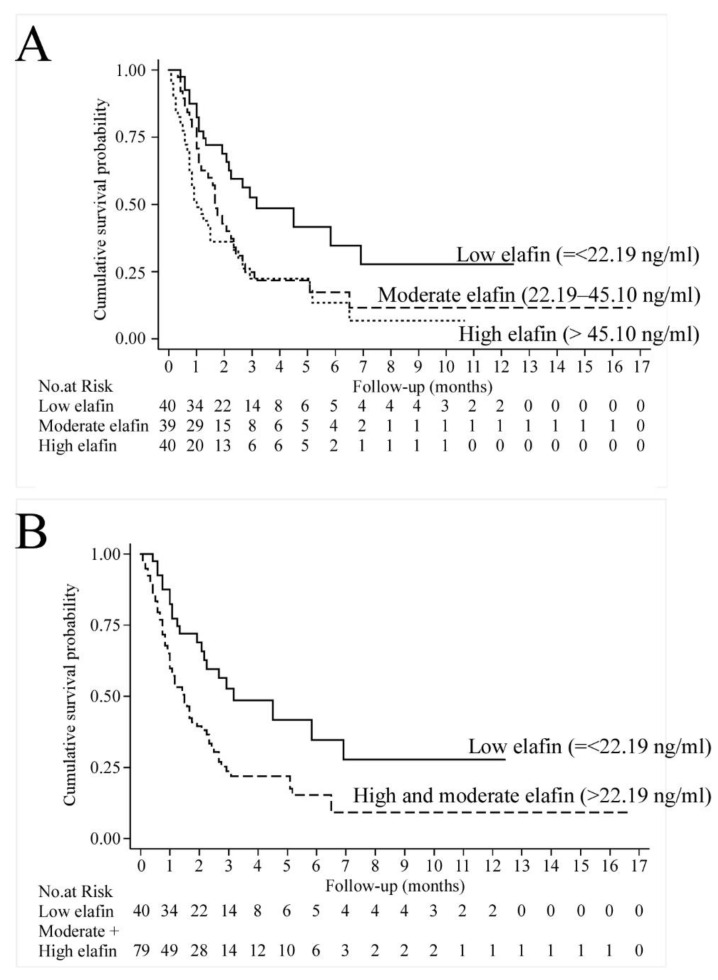
High levels of serum elafin were associated with poor prognosis in 119 stage I–IIIA esophageal squamous cell carcinoma patients. (**A**) Kaplan–Meier analysis of serum levels divided by tertile cut-off points (Log-rank test, *p* = 0.004); (**B**) Kaplan–Meier analysis of serum levels dichotomized by 1owest tertile cut-off point (Log-rank test, *p* = 0.002).

Our Kaplan–Meier survival curve found the serum elafin level tertiles to have significantly different prognostic predictions (log-rank, *p* = 0.004) (Figure 1A). The results remained similar after combining second and third tertiles (log-rank, *p* = 0.002) (Figure 1B). After adjusting for other covariates (age, sex, stage, and treatment), we found the hazard ratios (HR) to be 1.829 for the second tertile (*p* = 0.044) and 1.952 for the third tertile *p* = 0.032), compared to the first (Table 3). The combined second and third tertiles had an adjusted HR of 1.882 (*p* = 0.021), compared to the first (Table 3). We did not find any significant correlations of T and N with serum elafin levels (Supplementary Table S1). In addition, stage and treatment did not affect the significant effect of serum elafin levels on the prognosis of ESCC patients.

### 3.2. In Vitro Study

**Figure 2:**
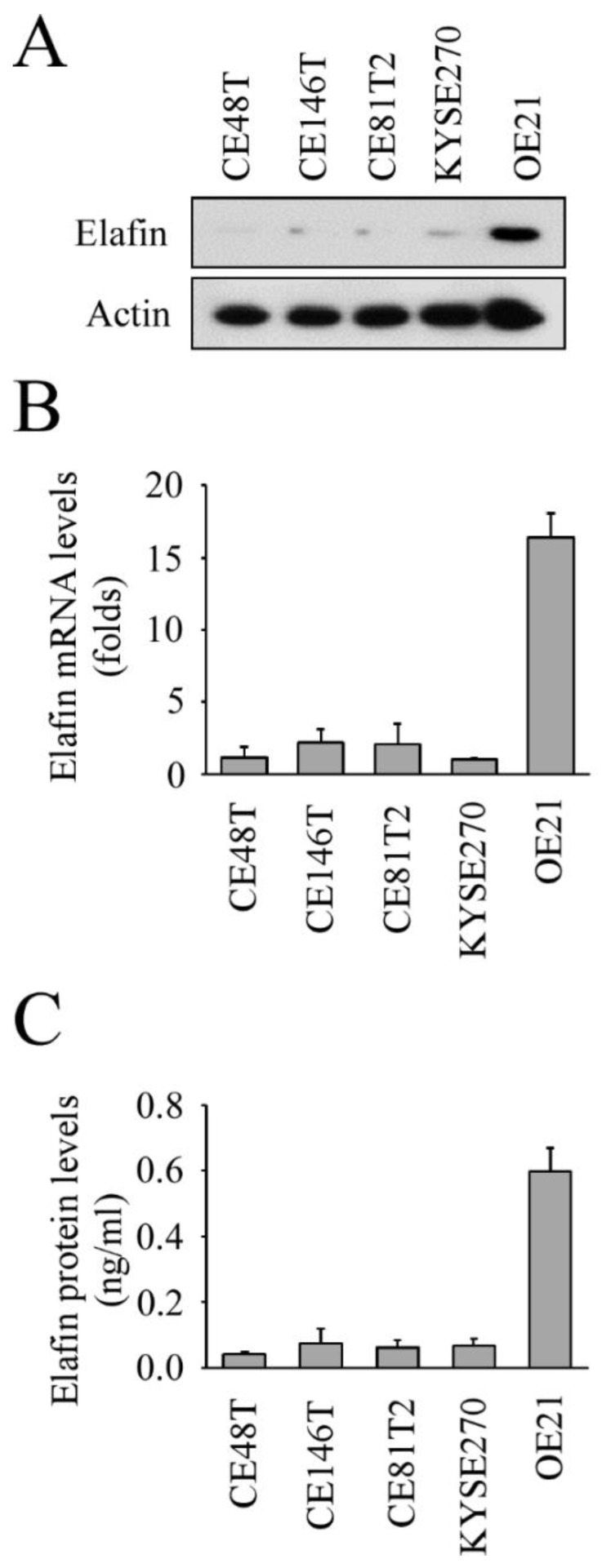
Expression and secretion of elafin in different esophageal squamous cell carcinoma cell lines: (**A**) the expression of elafin protein; (**B**) elafin mRNA expression levels; (**C**) the expression of elafin protein in cultured medium.

For our in vitro studies, we first measured elafin mRNA and protein levels in five different ESCC cell lines and found that OE21 had the highest intracellular and extracellular expression levels of elafin (Figure 2; Supplementary Figures S1 and S3). We then chose OE21 and CE81T2 to perform a subsequent elafin functional study.

**Figure 3:**
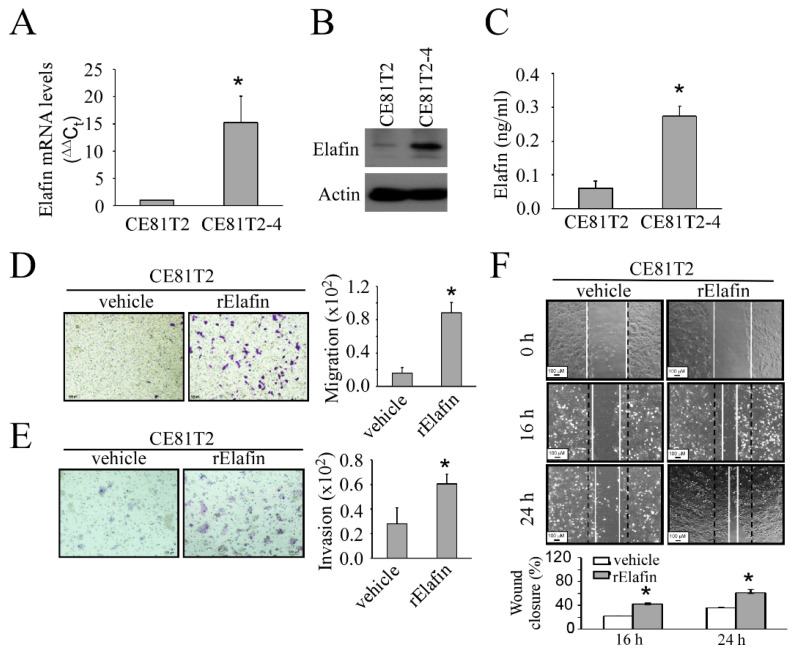
Elafin expression and its function in CE81T2 and its subpopulation (CE812-4) cell lines: (**A**) elafin mRNA expression level by qPCR in CE81T2 and CE81T2-4 cell lines; (**B**) elafin protein expression level by western blot in CE81T2 and CE81T2-4 cell lines; (**C**) secreted elafin protein level in supernatant of CE81T2 and CE81T2-4 cell lines; (**D**) increased migration ability of CE81T2 cell line after treatment with recombinant Elafin (rElafin) protein; (**E**) increased invasion ability of CE81T2 cell line after treatment with rElafin; (**F**) increased wound healing ability of CE81T2 cell line after treatment with rElafin. * indicates *p* < 0.05 when compared to the relative control.

In a previous study, we used a transwell invasion chamber to select different invasive subpopulations of the CE81T2 cell line and found that the CE81T2-4 cell line, which was the fourth round of sublines selected by BD BioCoat™ Matrigel™ Invasion Chamber (Franklin Lakes, NJ, USA), had the highest migration and invasion ability compared to the parental cell line (CE81T2) and the second-round subline (CE81T-2) [11]. In the current study, CE81T2-4 had higher elafin mRNA and protein expression than CE81T2, confirmed by qPCR and Western blot (Figure 3A,B; Supplementary Figure S3). The secreted elafin level was also found to be increased in the CE81T2-4 culture medium (Figure 3C). Treatment of recombinant elafin (rElafin) significantly increased the in vitro migration and invasion of CE81T2 (Figure 3D,E). In vitro wound-healing assay also showed that treatment of rElafin increased the directional migration of CE81T2 (Figure 3F).

**Figure 4:**
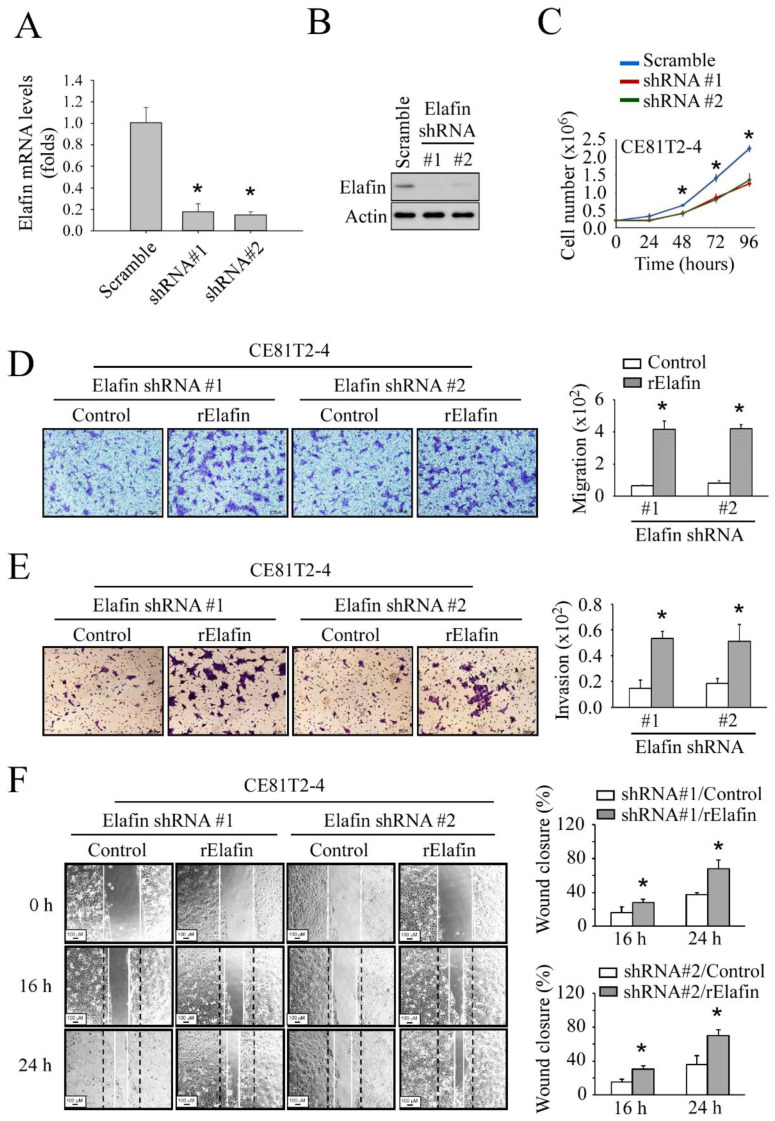
Elafin-specific shRNA suppressed the motility of the CE81T2-4 cell line and rescued by treatment of recombinant Elafin (rElafin) protein: (**A**) suppressed of elafin levels by elafin-specific shRNAs in CE81T2-4; (**B**) suppressed of elafin protein level by elafin-specific shRNAs in CE81T2-4; (**C**) knockdown elafin decreased cell proliferation in the CE81T2-4 cell line; (**D**) rescue migration ability of elafin-knockdown in the CE81T2-4 cell line after treatment with rElafin; (**E**) rescue invasion ability of elafin-knockdown in the CE81T2-4 cell line after treatment with rElafin; (**F**) rescued wound-healing ability of elafin-knockdown in the CE81T2-4 cell line after treatment with rElafin. * indicates *p* < 0.05 when compared to the relative control.

To further confirm that secretory elafin increased the ESCC motility, we knocked down elafin expression in CE81T2-4 and OE21 cancer cell lines (Figure 4A,B; Supplementary Figure S2). The proliferation and cell aggressiveness, including invasion, migration, and wound healing ability, of both cell lines were reduced (Figure 4C–F; Supplementary Figure S1). However, the cell aggressiveness of the CE81T2-4 cell line recovered after adding rElafin to the culture medium (Figure 4D–F). These findings suggest that both endogenous and secretory elafin promote ESCC progression and invasion.

## 4. Discussion

Some studies have found that elafin can drive poor prognosis in patients with breast and ovarian cancers [12,13,14], but there has been no study of its effect on ESCC prognosis, and only a very few studies have examined its role on ESCC risk [5]. We found that high serum elafin levels could predict a poor prognosis in early ESCC patients without distant metastasis (stage I-IIIA ESCC). Consistent with our clinical findings, we also found that elafin increased motility and invasion in ESCC cell lines.

Elafin, a secretory small molecule, is mainly involved in anti-microbial and anti-inflammatory functions [15,16,17]. Similar to secretory leukocyte proteinase inhibitor (SLPI), elafin belongs to the whey acidic protein family [18]. In 1985, elafin was first isolated from human bronchial secretions [19]. Additionally, expressed by macrophages and neutrophils, it acts as a multifunctional protein against the serine proteases and has immunomodulatory effects [20]. Many studies have found that elafin can play either an oncogenic role or a tumor-suppressive role in various cancers, depending on the different tissue/organ-specific entity [21,22]. In our review of its clinical role in cancer in the literature, we found that up-regulation of elafin has been associated with most cancers, including the brain, prostate, and squamous cell carcinoma phenotype of the lung, head and neck, and esophagus [6,10,23,24]. Its down-regulation has been found in breast cancer, and melanoma, probably basal cell carcinoma [12,25]. Both up- and down-regulation of elafin have been reported for ovarian cancer [12,23].

Alkemade and colleagues, studying the expression of elafin in ESCC, found it to be immunohistochemically overexpressed in tissues of squamous cell carcinoma and actinic keratosis, a pre-squamous cell carcinoma phenotype, but not expressed in basal cell carcinoma [26]. However, in that study, the tissue origin of squamous cell carcinoma (SCC) was unknown. Subsequently, Yamamoto et al., studying elafin expression in 34 ESCC patients, found that 25 (73.5%) of them collected overexpressed elafin, compared to normal mucosa of the esophagus [5]. However, the expression of elafin has been found to be stronger in well-differentiated ESCC patients than in poorly-differentiated ones, suggesting it may be involved in cell differentiation and apoptosis of squamous cell carcinoma cells of the esophagus [5]. Because the sample size of that cross-sectional pathological study was relatively small (*n* = 27), its results should be interpreted with some caution.

To the best of our knowledge, no previous study has examined the role of elafin on the prognosis of ESCC [27,28]. This study was the first one. However, one limitation of our study is we did not have detailed information about tumor grading and pathological reports of lymphatic and microvascular invasion, which may confound the prediction of serum elafin levels on the prognosis of ESCC patients (Supplementary Table S2). Another limitation is the lack of elafin expressions on targeted tissues, which are necessary to explore in the future study.

A few studies have reported high elafin expressions in tumors to be correlated with poor prognosis of breast cancer [12,29], ovarian cancer [23] and glioblastoma [24]. Elafin might serve as a double-edged sword in breast cancer in that it has been found to suppress the development of breast cancer on the one hand, while on the other hand, it has been found to promote its migration and invasion [12,13,14]. The detailed mechanisms underlying elafin regulation of cellular physiology are complex, and its role in the progression of cancer is controversial. Elafin can inhibit neutrophil elastase-induced cell growth of human mammary epithelial cells, a process known to be mediated by ERK signaling [30]. However, elafin also promotes cell proliferation through its activation of the MAP kinase pathway and has also been associated with chemoresistance via Bcl-Xl expression in high-grade serous ovarian cancers and basal-like breast tumors [13]. In SKOV3 ovarian cancer cells, knocking down elafin increases apoptosis after cisplatin treatment, and its overexpression leads to increased cisplatin resistance [31]. In our current in vitro study, we found that treatment with rElafin provoked proliferation, migration, and invasion of ESCC cell lines, whereas knockdown elafin had the opposite effect. Further mechanistic studies are needed to clarify how elafin regulates these activities in esophageal squamous tumor cells.

## 5. Conclusions

In summary, our human study and in vitro study found that elafin predicted the prognosis of ESCC and affected its aggressiveness. We conclude that serum elafin levels might potentially be used as a noninvasive marker able to predict survival of locoregional ESCC and could be potentially targeted to treat ESCC in some patients.

## Figures and Tables

**Table 1:** Baseline characteristics of the 119 stage I-IIIA ESCC patients.

No. of Patients	*n* = 119
Characteristics	Mean ± SD (range) or No. (%)
Gender	
Male	111 (95.3)
Female	8 (4.6)
Age (years)	57.83 ± 10.49 (38–82)
Alcohol	
No	16 (13.4)
Yes	103 (86.6)
Smoke	
No	11 (9.2)
Yes	108 (90.8)
Betel nut	
No	57 (47.9)
Yes	62(52.1)
Stage	
I	17 (14.3)
II	85 (71.4)
IIIA	17 (14.3)
Treatment	
CCRT + surgery	48 (40.3)
CCRT only	41 (59.6)
Serum elafin level (ng/mL)	40.14 ± 40.29 (0.59–367.89)

Abbreviation: ESCC, esophageal squamous cell carcinoma; CCRT, concurrent chemoradiotherapy.

**Table 2:** A comparison of demographic and clinical parameters categorized by tertile of serum elafin levels.

Serum Elafin Levels (ng/mL)	Low Elafin (*n* = 40) 0.59–22.19	Moderate Elafin (*n* = 39) 22.19–45.10	High Elafin (*n* = 40) 45.10–367.89	p *
**Characteristics**	**Mean ± SD or No. (%)**			
Age, years	54.64 ± 9.43	58.09 ± 10.81	60.78 ± 10.56	0.030
Gender (%, men)	37 (92.5)	36 (92.3)	38 (95.0)	0.867
Alcohol (%, yes)	37 (92.5)	31 (79.4)	31 (77.5)	0.232
Smoke (%, yes)	37 (92.5)	33 (84.6)	38 (95.0)	0.252
Betel nut (%, yes)	22 (55.0)	21 (53.8)	19 (47.5)	0.770
Stage				0.006
I	10 (25.0)	3 (7.7)	4 (10.0)	
II	20 (50.0)	31 (79.4)	34 (85.0)	
IIIA	10 (25.0)	5 (12.8)	2 (5.0)	
Treatment				<0.001
CCRT + surgery	26 (65.0)	11 (28.2)	11 (27.5)	
CCRT only	14 (35.0)	28 (71.8)	29 (72.5)	

Abbreviation: CCRT, concurrent chemoradiotherapy. * One-way ANOVA or Chi-square test.

**Table 3:** Hazard ratio (HR) of 119 ESCC patients categorized by tertile of serum elafin levels.

Cut-Off Point	*n*	Unadjusted		Risk-Adjusted *		Risk-Adjusted **	
		HR (95% CI)	*p*	HR (95% CI)	*p*	HR (95% CI)	*p*
Tertile							
Low	40	1		**1**		**1**	
Moderate	39	1.88 (1.07, 3.28)	0.028	1.96 * **(1.10, 3.47)**	**0.022**	1.88 ** **(1.04, 3.37)**	**0.035**
High	40	2.47 (1.42, 4.29)	0.001	2.22 * **(1.24, 3.97)**	**0.007**	2.13 ** **(1.06, 3.88)**	**0.013**
Dichotomize							
Low	40	1		**1**		**1**	
Moderate + High	79	2.14 (1.30, 3.52)	0.003	2.08 * **(1.24, 3.47)**	**0.005**	1.99 ** **(1.17, 3.38)**	**0.011**

Abbreviation: ESCC, esophageal squamous cell carcinoma. * Adjusted risk factors for age, sex, and treatment. ** Adjusted risk factors for age, sex, stage, and treatment. The bold value indicates statistical significance.

## Data Availability

The data presented in this study are available on request from the corresponding author.

## Supplementary Materials

The following are available online at https://www.mdpi.com/article/10.3390/cancers13123082/s1, Figure S1: Western blot quantification, Figure S2: Elafin-specific shRNA suppressed the motility of the OE21 cell line, Figure S3. Uncropped Western Blot images, Table S1: Comparison of demographic and TNM categorized by tertile of serum elafin levels, Table S2: Hazard Ratio (HR) of 119 ESCC patients in a Cox regression model.
