# Supplementary material for: High Serum Elafin Prediction of Poor Prognosis of Locoregional Esophageal Squamous Cell Carcinoma
Source: Cancers (Basel). 2021 Jun 21;13(12):3082. doi: 10.3390/cancers13123082 (PMC8233752; doi:10.3390/cancers13123082)
Supplement: Supplementary file 1 [file cancers-13-03082-s001.zip › cancers-1213852-supplementary.pdf]

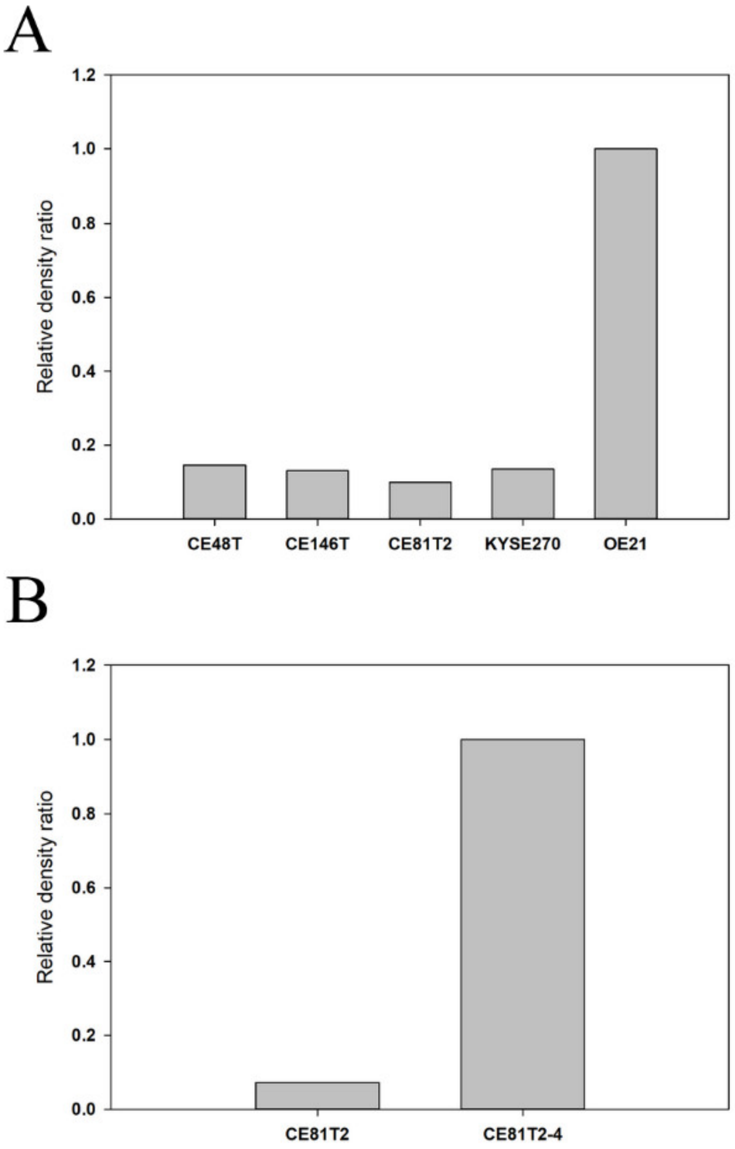

Figure S1. The western blot quantification. The signal of each protein was quantified by Gel analysis function of Image J, and calculated the relative density ratio after normalizing with internal control. (A) Quantification data of figure 2A (B) Quantification data of figure 3B

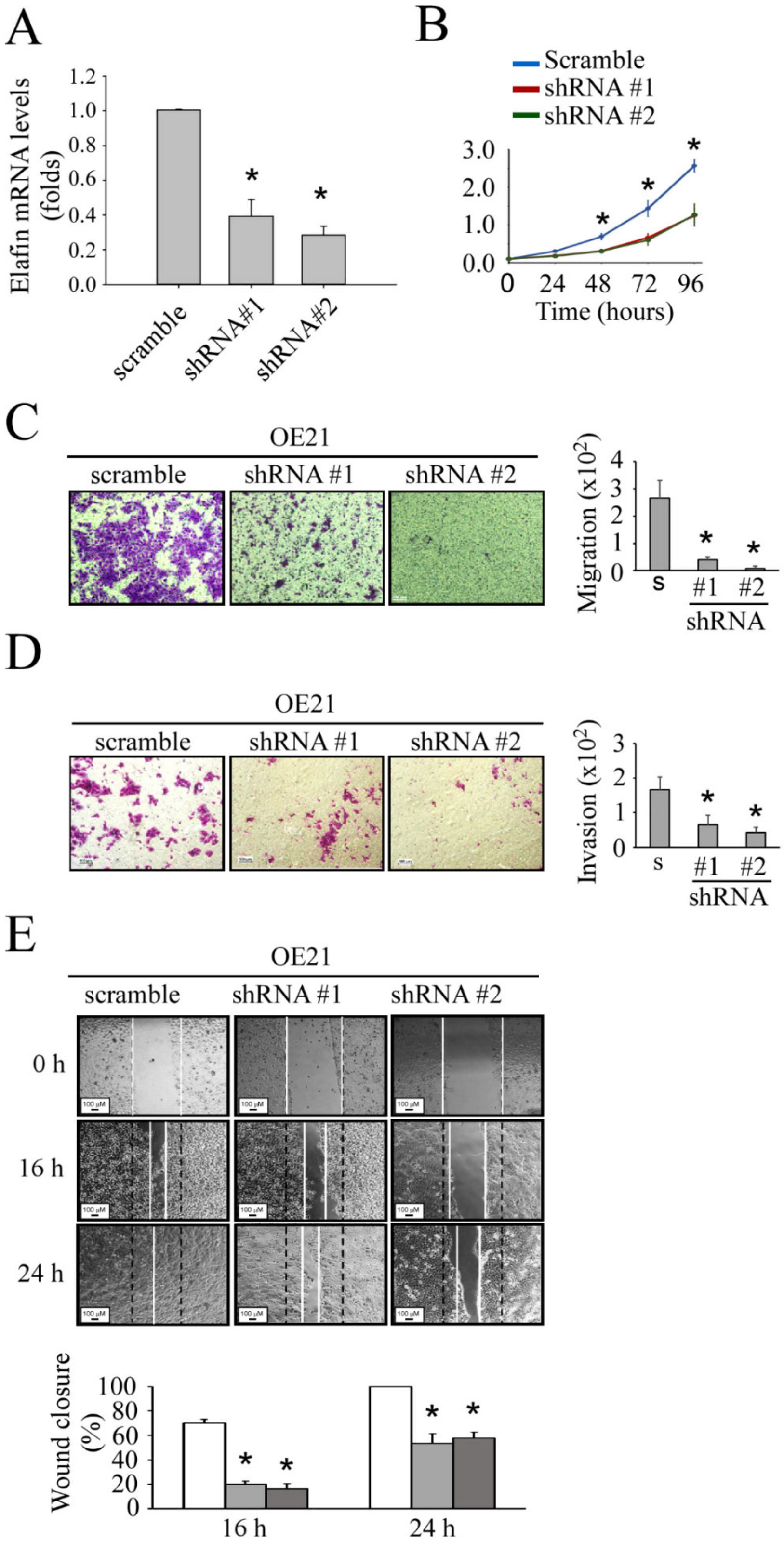

Figure S2. Elafin-specific shRNA suppressed the motility of OE21 cell line. (A) Suppressed elafin levels by elafin-specific shRNAs in OE21; (B) Knockdown elafin

decreased cell proliferation in OE21; (C) Knockdown elafin decreased cell migration in OE21; (D) Knockdown elafin decreased cell invasion in OE21; (E) Knockdown elafin decreased cell wound healing in OE21. * indicates $P<0.05$, when compared to the relative control.

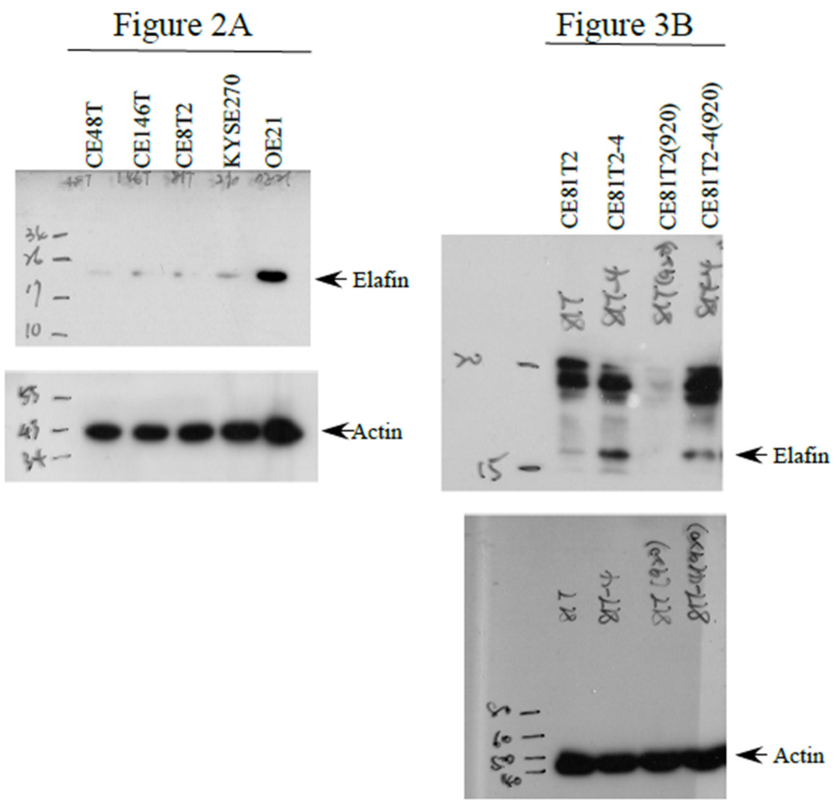

Figure S3. Uncropped Western Blot images.

Table S1. Comparison of demographic and TNM categorized by tertile of serum elafin levels.

Serum elafin levels (ng/ml)	Low elafin 0.589-22.19 (n=40)	Moderate elafin 22.19-45.10 (n=39)	High elafin 45.10-367.89 (n=40)	*P**
**Characteristics+**		**No. (%)**		
Tumor				0.218
T1	8 (20.0)	4 (10.3)	5 (12.5)	
T2	15 (37.5)	17(43.6)	24 (60.0)	
T3+T4a	17 (42.5)	18(46.2)	11 (27.5)	
Node				0.829
N0	26 (65.0)	29 (74.4)	25 (62.5)	
N1	13 (32.5)	9 (23.1)	14 (35.0)	
N2	1 (2.5)	1 (2.6)	1 (2.5)	

*Chi-square test.

+According to the American Joint Committee on Cancer (AJCC) 7th edition of the tumor-node-metastasis (TNM) cancer staging system. None of our study subjects had distant metastasis.

Table S2. Hazard Ratio (HR) of 119 ESCC patients in a Cox regression model.

Cut-off point	n	Unadjusted		Risk-adjusted*	
		HR (95% CI)	p	HR (95% CI)	p
**Age, years**	119	1.014 (0.993, 1.035)	0.199	**1.011 (0.987, 1.035)**	**0.371**
**Gender**					
Male	111	1		**1**	
Female	8	0.340 (0.122, 0.947)	0.039	**0.296 (0.103, 0.850)**	**0.024**
**Ealfin by tertile**					
Low	40	1		**1**	
Moderate	39	1.88 (1.07, 3.28)	0.028	**1.88** (1.04, 3.37)**	**0.035**
High	40	2.47 (1.42, 4.29)	0.001	**2.13 (1.06, 3.88)**	**0.013**
**Stage**					
I	17	0.746 (0.382, 1.455)	0.390	**0.877 (0.436, 1.762)**	**0.712**
II	85	1		**1**	
IIIA	17	0.733 (0.364, 1.476)	0.384	**0.757 (0.370, 1.548)**	**0.446**
**Treatment**					
CCRT only	71	1		**1**	
CCRT + surgery	48	0.810 (0.515-1.276)	0.363	**1.016 (0.622, 1.659)**	**0.949**

*Variables of age, sex, stage, treatment, and serum elafin levels in the multivariate model.
